# Locally advanced breast cancer: primary ultra-hypofractionated radiotherapy for inoperable or frail patients

**DOI:** 10.1007/s00066-025-02445-5

**Published:** 2025-09-23

**Authors:** Anne Caroline Knöchelmann, Roland Merten, Hans Christiansen, Elna Kuehnle, Daniela Meinecke

**Affiliations:** 1 Department of Radiotherapy, Hannover Medical School, Carl-Neuberg-Str. 1, 30625 Hannover, Germany; 2 Departments of Gynaecology and Obstetrics, Hannover Medical School, Carl-Neuberg-Str. 1, 30625 Hannover, Germany

**Keywords:** Breast cancer, Radiotherapy, Hypofractionated, Organ preservation, Palliative care

## Abstract

**Purpose:**

Locally advanced breast cancer in frail and inoperable patients often causes tumor-associated pain, bleeding, or discharge. These patients may not be suitable for therapeutic options like surgery or potentially toxic systemic treatment. Local radiotherapy with little impact on treatment time may be beneficial in this patient subgroup. We evaluated an ultra-hypofractionated definitive irradiation concept in five fractions (5 × 5 Gy with a simultaneous integrated boost of 5 × 6 Gy) for these patients, focusing on tolerability and clinical outcome.

**Methods:**

A total of 29 patients were retrospectively sampled. They were treated by irradiation to the breast with 25 Gy in five fractions with a simultaneous integrated boost (SIB) of 6 Gy per fraction. Tumor response and clinical outcome were evaluated by clinical examination.

**Results:**

In total, 27 patients with a median age of 82 years were assessed. Median follow-up was 7.4 months. All patients completed radiotherapy with 25 Gy in five fractions with a simultaneous integrated boost of 30 Gy (6 Gy per fraction) without any high-grade toxicity (≥ grade 2). Within the first 90 days after irradiation, 15 patients (56%) exhibited a clinical response and 12 showed stable disease. Only 7 patients reported low-grade acute dermatotoxicity grade 1 (CTCAE) within the first 90 days, and only one experienced toxicity later (fibrosis grade 1, LENT-SOMA).

**Conclusion:**

Radiotherapy in five consecutive daily fractions is sufficient. The studied regimen proved to be a safe, effective palliative treatment in inoperable and frail patients not suitable for surgery or toxic systemic therapy.

## Introduction

Locally advanced breast cancer is a challenging situation, demanding multimodal treatment. In cases of operable disease, neoadjuvant systemic treatment is a standard approach focused on downstaging and in vivo drug sensitivity testing [1]. For patients diagnosed in an advanced stage, who cannot be provided with systemic treatment or surgery due to a reduced general condition and/or comorbidities, the treatment focus is based on avoiding possible local complications such as ulceration, bleeding, or pain, when anti-hormonal therapy in receptor-positive patients alone is not successful or does not promise a rapid onset of effect.

In the past two decades, numerous hypofractionated regimens have been established in the adjuvant treatment setting to determine the optimal treatment schedule of radiation therapy in breast cancer. To our knowledge, these trials were based on patient collectives treated in curative settings after surgery. In these cases, adjuvant radiotherapy was delivered to reduce the risk of local relapse. Hypofractionated radiotherapy regimens have been established as the standard of care; for example, the Ontario and START‑B trials found hypofractionated radiotherapy in an adjuvant setting in 15–16 fractions delivered over 21–22 days to be effective and well tolerated [2, 3]. The UK FAST trial showed that 5.7 or 6.0 Gy delivered once weekly is radiobiologically equivalent to 50 Gy delivered in 25 fractions. Concerning the treatment time, the 5‑year results of the UK Fast-Forward trial proved 26 Gy in 5 fractions delivered over one week to be as effective as 40 Gy delivered in 15 fractions over 3 weeks for local tumor control. Normal tissue effects up to 5 years were similar for the 40-Gy (15 fractions) and 26-Gy (5 fractions) schedules [4, 5]. Thus, the high tolerability of hypofractionation has already been demonstrated in the context of curative radiotherapy of the breast.

Although locally advanced inoperable disease is common in the elderly population, few data exist on treatment options, clinical characteristics, and outcomes after hypofractionated radiotherapy [6]. As the incidence of patients with locally advanced breast cancer who cannot undergo regular treatment schedules due to frailty is increasing, there is a need for highly individualized treatment concepts with short overall treatment times and minimal side effects on normal tissue [7–9]. High-dose definitive radiotherapy such as ultra-hypofractionated concepts have been proven to be well tolerated in patient cohorts with unresectable locally advanced breast cancer and to provide good symptom control concerning bleeding [10–12].

In this retrospective single-center study, we report the results of a short-duration ultra-hypofractionated treatment schedule with external beam radiotherapy as a safe and effective option for patients suffering from breast cancer with symptoms like pain, bleeding, and ulceration.

## Methods

### Patient selection

From January 2020 to November 2024, a total of 29 patients were identified in our institution who were treated with local radiotherapy for locally advanced breast cancer without surgery using an ultra-hypofractionated treatment regimen. Two patients were excluded: one due to histology (angiosarcoma) and one who cancelled radiotherapy after two fractions due to worsening general condition. All data on patient demographics, tumor characteristics, and radiotherapy parameters as well as regarding oncological response were collected from the patients’ charts. The treatment concept was approved prior to treatment by a multidisciplinary tumor board.

### Radiotherapy

Radiotherapy was delivered as external beam linac-based irradiation with 6‑MV photons. All patients were positioned supine on breast boards with both arms overhead. In all cases, a CT scan with a 3-mm secondary reconstruction interval was performed prior to treatment. Volumetric intensity-modulated arc therapy (VMAT) was used, with a bolus applied to the tumor region in case of skin involvement of the tumor or ulceration. Dosimetry was performed according to the International Commission on Radiation Units and Measurements (ICRU) report No. 83 [13] using three-dimensional dose optimization with the Elekta Monaco algorithm (Elekta, Stockholm, Sweden).

The patients in our collective were treated with 25 Gy in five fractions, with a simultaneous integrated boost up to 30 Gy total dose in five fractions. Due to the palliative intention, expansion of the target volume was determined individually based on the planning CT. The clinical target volume (CTV) of the 5‑Gy volume comprised the entire breast as well as the adjacent lymphatic drainage and any cutaneous infiltration in the case of tumor invasion. To create the planning target volume (PTV), a margin of 6–8 mm was added; this volume, including the entire breast as well as the adjacent lymphatic drainage, was irradiated with a single dose of 5 Gy. An additional boost of 1 Gy per fraction (cumulative boost dose corresponding to 30 Gy in 5 fractions) was integrated using macroscopic disease within the breast (gross target volume, GTV), surrounded by a margin of 3 mm to create the CTV and a further 3 mm to create the PTV. This volume comprised only the macroscopic primary tumor in the breast, with no lymph nodes involved, and was irradiated as a simultaneous integrated boost (SIB) volume with a cumulative single dose of 6 Gy per fraction.

A cone-beam CT scan was performed before each fraction for image-guided radiotherapy (IGRT).

### Patients

A total of 27 eligible patients were identified and treated with palliative ultra-hypofractionated radiotherapy for locally advanced breast cancer in our institute between January 2020 and November 2024. One had been treated with radiotherapy to the same target volume (ipsilateral breast with a conventionally fractionated irradiation dose of 50.4 Gy to the entire breast) 5 years prior and three had been treated with additional radiotherapy beyond the current target volume (thoracic spine due to painful metastasis, whole-brain irradiation due to cerebral metastasis, kidney due to renal metastasis).

Patient characteristics are shown in Table 1. The median age of the patients was 82 years (range 28–96 years). Due to hormone receptor positivity (77.8% of patients), most patients received endocrine therapy upfront (74.1%). A total of 14 patients (51.9%) showed distant metastasis at the date of presentation.

**Table 1:** Patient and tumor characteristics

Characteristic	*n* (%)
*Median age in years (range)*	82 (28–96)
*ECOG*	
0	3 (11.1%)
1	9 (33.3%)
2	6 (22.2%)
3	7 (25.9%)
4	2 (7.4%)
*Initial T stage*	
T1	0 (0.0%)
T2	4 (14.8%)
T3	2 (7.4%)
T4	21 (77.8%)
*Initial N stage*	
N0	10 (37.0%)
N1	15 (55.6%)
N2	2 (7.4%)
*Initial M stage*	
M0	13 (48.1%)
M1	14 (51.9%)
*Grading*	
1	2 (7.4%)
2	14 (51.9%)
3	11 (40.7%)
*Hormone receptor status*	
Positive	21 (77.8%)
Negative	6 (22.2%)
*Her2 status*	
Positive	2 (7.4%)
Negative	25 (92.6%)
*Initial systemic treatment*	
Endocrine therapy	20 (74.1%)
CDK4/6 inhibitor	6 (22.2%)
Other systemic treatment	5 (18.5%)

*EGOG* Eastern Cooperative Oncology Group performance status, *T* tumor size; *N* nodal status; *M* metastasis

### Radiotherapy details

Details of radiotherapy are shown in Table 2. The mean irradiated volume of the entire breast including the lymphatic tissue in the case of tumor (PTV1.0) was 1132.5 ± 689.9 ml (97.4–2784.0 ml), and the PTV of the boost to the primary tumor (PTV1.1) was 238.2 ± 387.1 ml (13.2–1910.8 ml). A total of 12 patients were irradiated with 5 mm of local bolus in the case of ulceration to increase the radiation dose to the skin surface in the area of exulceration. The mean dose to the heart was 1.4 Gy, the mean volume of the ipsilateral lung irradiated with 13 Gy (V_13Gy_) was 9.9%, and the mean volume of the ipsilateral lung irradiated with 5 Gy (V_5Gy_) was 27.9%. The ipsilateral brachial plexus received a maximum dose of 11 Gy.

**Table 2:** Radiotherapy details

	Mean ± SD	Range
PTV 1.0 (ml)	1020.3 ± 678.3	97.4–2784.0
PTV 1.1 (ml)	213.7 ± 354.7	13.2–1910.8
Heart D_mean_ (Gy)	1.4 ± 0.7	0.2–3.3
Lung V_13Gy_ (%)	9.9 ± 5.4	0.0–20.0
Lung V_5Gy_ (%)	27.9 ± 16.0	2.0–74.5
Plexus brachialis D_max_ (Gy)	11.0 ± 10.9	0.1–26.2
*Bolus*	*n*	*Percentage*
Yes	12	44.4
No	15	55.6

*PTV* planning target volume, *V*_*13Gy*_ irradiated volume in percent with 13 Gy, *V*_*5Gy*_ irradiated volume in percent with 5 Gy, *D*_*max*_ maximum dose, *SD* standard deviation

**Fig. 1:**
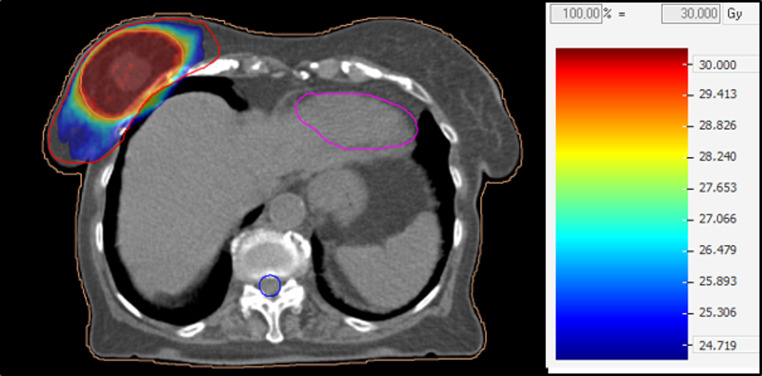
Radiation treatment plan. Red contour = PTV 1.0 of the entire breast; orange contour = PTV 1.1 of the simultaneous integrated boost; isolines of radiation beams are in Gray (color wash; 30 Gy = 100% of the total dose). *PTV* planning target volume

Figure 1 shows an example of a radiation treatment plan that delivers a homogeneous dose to the entire breast volume with a simultaneous integrated boost to the primary macroscopic tumor.

### Follow-up

Local control was defined as clinical response or the absence of progression and was measured from the last application of RT until the first clinical examination within 90 days after completing radiotherapy. The last follow-up was defined as the last clinical examination by the treating oncologist or gynecologist or the last imaging diagnostics performed to evaluate treatment response and the residual extent of disease. Adverse side effects were evaluated as grade 0 to 4 according to the Common Terminology Criteria for Adverse Events (CTCAE) version 5.0 for acute effects [14] and according to LENT-SOMA (Late Effects on Normal Tissue/Subjektive, Objective Management and Analytic) [15] for late effects. Due to age, frailty, or immobility, many patients were treated with the intention of providing palliation and either came for only one follow-up examination or not at all.

Consequently, the findings of the retrospective data analysis are limited to a single follow-up examination. This restricts the ability to detect late effects or re-progression in the context of their aftercare due to loss to follow-up.

## Results

### Clinical outcome (local control, clinical response)

Table 3 shows local symptoms prior to radiation therapy as well as treatment times. The median time between the date of first diagnosis and the first day of radiotherapy was 360 days, with a range of 14 to 1435 days. Most patients included in the study suffered from local tumor ulceration (20 patients; 74.1%). Further harmful symptoms were secretion (9 patients; 33.3%), pain (7 patients; 25.9%), and local bleeding (6 patients; 22.2%). The overall radiotherapy treatment time between the first and the last irradiation was 10.7 days (range 7–16 days).

**Table 3:** Local symptoms, radiation treatment times, and initial local response

**Initial local symptoms**	
Bleeding	6 (22.2%)
Exulceration	20 (74.1%)
Pain	7 (25.9%)
Secretion	9 (33.3%)
Oedema	4 (14.8%)
*Time between ED and first RT (days)*	
Mean ± SD (min–max)	390 ± 378 (14–1435)
*First RT to last RT (days)*	
Mean ± SD (min–max)	10.7 ± 2.3 (7.0–16.0)
**Initial local response**	
*Regression (n)*	15 (55.6%)
Regression after 90 days (*n*)	11
Progression after 90 days (*n*)	1
LTFU	3
*Stable disease (n)*	12 (44.4%)
Regression after 90 days (*n*)	6
Progression after 90 days (*n*)	2
LTFU	4
*Progression*	0

*ED* date of initial diagnosis; *RT* radiotherapy; *SD* standard deviation; *n* number of patients; *LTFU* lost to follow-up

Directly after completion of radiotherapy, no progress was documented in any case. Most patients experienced local remission (*n* = 15; 55.6%), while the others at least showed stable disease (*n* = 12; 44.4%).

Of those who had an initial local remission, 11 patients continued to have local control after 90 days, while one patient suffered local progression 90 days after completion of radiotherapy (this patient showed an aggressive tumor biology with a triple-negative, G3 tumor). A total of 3 patients from this group were lost to follow-up.

Of the 12 patients (44.4%) with stable disease after completion of radiotherapy, 6 showed a delayed local remission after 90 days, and 2 showed local progression after 90 days. In this group, 4 patients were lost to follow-up.

The evaluation of responses is constrained in this retrospective analysis because most patients only attended a single aftercare appointment, due to factors such as advanced age, mobility restrictions, and frailty. Consequently, late effects or re-progression were not identified within the framework of their aftercare due to loss to follow-up.

**Fig. 2:**
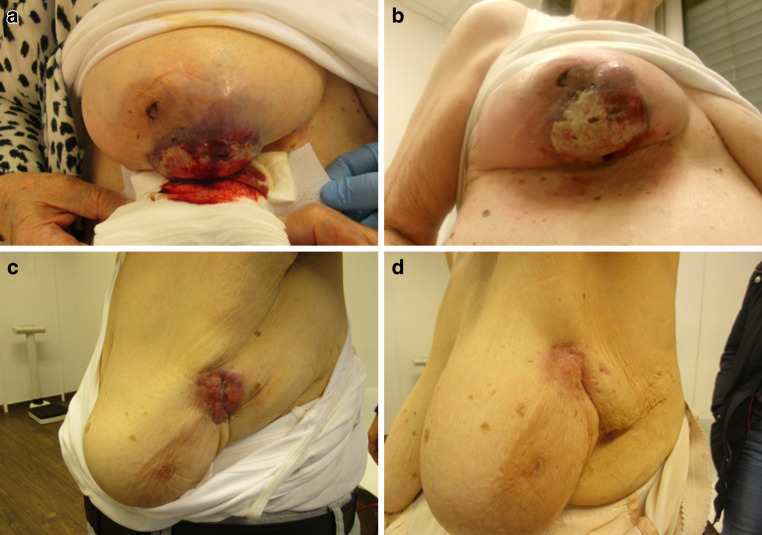
Local effect in the sense of a response to therapy. **a** Patient with bleeding and ulceration due to expansive tumor before initiating radiotherapy; **b** clinical response and sufficient hemostasis on the last day of radiotherapy. **c** Patient with painful and expansive tumor before initiating radiotherapy; **d** local remission 5 months after completion of radiotherapy

In cases of bleeding and local ulceration, local radiotherapy can have a visible effect, with an improvement in symptoms as early as the last day of radiotherapy. Figure 2 shows the example of a patient who experienced a significant improvement in symptoms on the last day of radiotherapy as well as an example of the late effect of local irradiation, with local remission 5 months after the last day of irradiation.

**Fig. 3:**
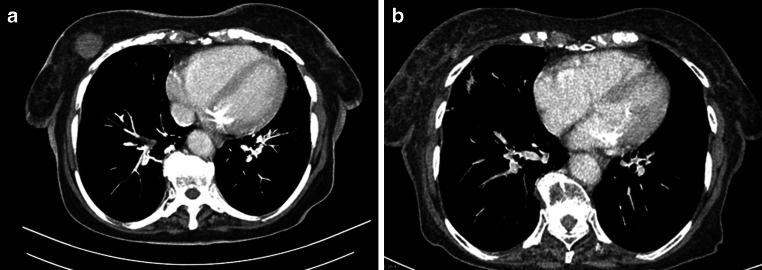
Local tumor remission on CT imaging: **a** before radiotherapy, **b** 3 months after radiotherapy. *CT* computer tomography

Figure 3 shows another patient’s good clinical response using CT imaging.

### Acute/late toxicity

The clinical examination on the last day of radiotherapy showed that most patients (*n* = 20; 74.1%) had no local acute side effects in the surrounding area of non-ulcerated skin, and 7 patients (25.9%) had low-grade local radiodermatitis grade 1 with no need for medical treatment (Table 4). Only one patient showed local fibrosis on clinical gynecological follow-up 90 days after the last treatment. No grade 2–4 toxicities were observed. Late toxicity was determined through regular care by the treating gynecologist or oncologist as part of a clinical examination. All toxicities that persisted for over 90 days after the last treatment were defined as late. The mean interval between the last day of radiotherapy treatment and the last clinical follow-up examination to assess response and toxicity was 7.3 months (range 36–619 days). Some patients received clinical examinations sooner than 90 days after treatment, while other patients were followed up over a longer interval.

**Table 4:** Acute and late toxicity

Toxicity	*n* (%)
*Acute*	
Grade 0	20 (74.1%)
Grade 1	7 (25.9%)
Grade 2–4	0 (0.0%)
*Late*	
Grade 0	26 (96.3%)
Grade 1	1 (3.7%)
Grade 2–4	0 (0.0%)

Acute toxicity: grade according to the Common Terminology Criteria for Adverse Events (CTCAE) v5

Late toxicity: grade according to Late Effects of Normal Tissue/Subjective, Objective, Management, and Analytic (LENT-SOMA)

## Discussion

This ultra-hypofractionated irradiation schedule using five consecutive fractions for locally advanced breast cancer is well tolerated. In addition, a good clinical response was observed in almost all studied inoperable and/or frail patients. Adverse effects were rare, and almost all patients showed a good clinical response within the first 90 days after completion of local therapy.

While there is a need to develop hypofractionated radiotherapy concepts in a patient population with advanced tumor disease, the optimal regimen remains unclear [26]. The previous literature presents other experiences of hypofractionated palliative radiotherapy in locally advanced cancer and fungating wounds [29]. For example, palliative hemostatic radiotherapy was proven to be effective in the treatment of advanced pelvic gynecological malignancies. Meixner et al. investigated different fractionation schemes and observed a biologically effective dose (BED) cut-off value of BED_α/β_ _=_ _10_ = 36 Gy [25]. They reported high cessation of bleeding (80%) and good pain relief (60%) in their cohort. Furthermore, in non-melanoma skin cancer, hypofractionated radiation therapy was established to be effective and well tolerated, with a wide range in BED_α/β_ _=_ _10_ from 30 to over 157 Gy [28]. In addition, it has been found that in the treatment of pelvic malignancies of different tumor entities with gross hematuria, a BED_α/β_ _=_ _10_ ≥ 36 Gy is required for symptom relief [27]. In our fractionation scheme, we apply a BED_α/β_ _=_ _10_ = 37.5 Gy, and to offer relief to the surrounding organs at risk, we apply a BED_α/β_ _=_ _10_ = 45 Gy as a simultaneous integrated boost to the primary gross tumor volume.

Various hypofractionated radiation regimens have already been published for this patient population. An overview of studies with similar palliative radiotherapy concepts is shown in Table 5.

**Table 5:** Overview of studies with similar palliative radiotherapy concepts

Authors, year, center (ref.)	Radiation concept	Number of patients	Timepoints of assessment of local control and treatment toxicity	Local control	Toxicity
Choi H.S. et al., 2019, Seoul St. Mary’s Hospital, Seoul [16]	42.5–55 Gy with 2.5–3 Gy per fraction, 5 fractions per week	22	1 week after end of RT, every 2 months for the first year, every 6 months for the next 3 years (median FU 14 months)	81.8% PR; 13.6% SD; 4.5% PD	27.3% grade 1 skin dermatitis; 72.7% grade 2 skin dermatitis; no grade 3
Yee C. et al., 2018, Sunnybrook Health Sciences Center, Toronto [18]	<40 Gy with 2 Gy per fraction (EQD2; 8 Gy/1, 20 Gy/5, and 30 Gy/10 fractionation), 5 fractions per week	43	No fixed regimen for the follow-up examination (median FU 25 months)	16% CR; 67% PR; 2% SD; 12% PD	60% moist desquamation; no grade 4 toxicity
Courdi A. et al., 2006, Radiotherapy Department, Centre Antoine-Lacassagne [19]	32.5 Gy with 6.5 Gy per fraction, followed by 1–3 fraction for a boost, once a week	115	Every 4–6 months for the first 2 years; annually thereafter (median FU 41 months)	78% SD or PR; 22% PD	20% grade 1 dermatitis; 8% grade 2 dermatitis
Hoeltgen L. et al., 2023, University Hospitals of Heidelberg [20]	Different regimens with a median cumulative dose of 39 Gy (range 9–54) with 3 Gy (range 1.8–4) per fraction	26	No fixed regimen for the follow-up examination (median FU 6.5 months)	LC 75.0% (after 6 months) and 47.6% (after 12 months)	No high-grade toxicities have been reported (> CTCAE grade 2)
Nakamura N. et al., 2018, multi-institutional prospective observational study, Kashiwa [21]	30–39 Gy with 3 Gy per fraction; 50 Gy with 2.5 Gy per fraction; 60 Gy with 2 Gy per fraction	21	1, 3, and 6 months after radiotherapy	Bleeding/discharge and offensive odor were significantly improved after radiotherapy	5% grade 2 dermatitis; 10% grade 3 dermatitis
Webb K. et al. 2023, The Royal Marsden Hospital, London [22]	30–36 Gy with 6 Gy per fraction, 1–5 fractions per week	109	No fixed regimen for the follow-up examination (median FU 46 months)	10.3% CR; 76.9% PR; 11.5% SD; 1.3% PD	35.7% grade 1 dermatitis; 31.6% grade 2 dermatitis; 7.1% grade 3 dermatitis
Present study	30 Gy with 5 Gy per fraction with simultaneous integrated boost with 6 Gy to the macroscopic tumor, 2–5 fractions per week	27	Clinical examination 3 months after radiotherapy as part of the radiotherapy follow up (median FU 7.4 months)	63% regression; 11% PD; 26% Lost to follow up	25.9% grade 1 dermatitis

Acute toxicity: grade according to the Common Terminology Criteria for Adverse Events (CTCAE) v5

Late toxicity: grade according to Late Effects of Normal Tissue/Subjective, Objective, Management, and Analytic (LENT-SOMA)

*PR* partial remission, *CR* complete remission, *SD* stable disease, *PD* progressive disease, *LC* locoregional control, *FU* follow-up, *EQD2* equivalent dose in 2 gray fractions

The radiation concept shown in our study is completed within a very short overall treatment time (maximum treatment period of up to 2 weeks). The tolerability is excellent, with maximally grade 1 toxicity locally in the radiation field and no higher-grade toxicities, unlike other studies that have described grade 2 skin toxicity. Our data also show excellent local control rates compared to the other studies, with very little local progression after local radiation therapy (only 3% of patients showed local progression and no improvement in symptom burden).

In the palliative setting, it is important to shorten the overall treatment time for patients, to reduce transport times and hospital stays. Giving irradiation treatment twice to three times per week allows time for normal tissue to recover before repopulation of tumor cells, as shown in palliative treatment in other tumor entities [23].

As shown in Table 5, several schemes for fractionation have already been established in the field of palliative radiotherapy for advanced breast cancer. Choi et al. [16] established a radiation concept with total doses of up 42.5–55 Gy with 2.5–3 Gy per fraction once per day, 5 days a week. In this cohort, the authors noted good symptom relief and a strong response in terms of local tumor control (over 80% partial response or stable disease). Nevertheless, over 70% of the treated patients experienced grade 2 moist desquamation of the skin, which is a greater percentage than reported in our study. The overall treatment time in this cohort was 3–5.5 weeks. With our fractionation concept, patients are finished with radiotherapy in 2–3 weeks, which saves time and avoids delaying any systemic therapies that may be indicated. Hoeltgen et al. [20] analyzed different concepts with a wide range of doses: median BED 68.3 Gy (range 40.0–94.5) and EQD2 45.5 Gy (range 26.7–63.0). The cumulative RT dose most frequently used in this work was either 39 or 45 Gy with a single dose of 3 Gy. Symptom palliation was reported in 95% of patients with low-grade side effects, predominantly erythema and fatigue. This study describes effective concepts that can be further shortened with even stronger hypofractionation, as in our concept, with similar effectiveness and tolerability.

Similar to our fractionation concept, in other studies, patients were irradiated with single doses of 6 Gy (Webb et al. [22]) or 6.5 Gy (Courdi et al. [19]) in somewhat larger cohorts. Webb et al. [22] compared once-weekly versus accelerated fractionation schedules in their cohort with 6‑Gy fractions over 6 weeks, administering up to 30 Gy to the whole breast and involved lymph nodes with or without a 6-Gy boost to the affected tumor quadrant (total dose not exceeding 36 Gy). They reported no significant difference in the groups regarding the median time to local progression or toxicity, but one patient who received five fractions a week suffered from grade 4 late toxicity (skin radionecrosis). Courdi et al. [19] delivered 6.5 Gy up to a cumulative 32.5 Gy in five fractions to the whole breast, followed by 1–3 fractions of 6.5 Gy to the tumor site, once weekly. In this study, the 5‑year local progression-free rate was 78%, with a low rate of early reactions and 52% late effects, mainly subcutaneous fibrosis. To prevent grade 4 toxicity as reported by Webb and to prevent late effects like subcutaneous fibrosis, we established treatment breaks between the fractions in our concept, so that no patient was irradiated for five consecutive days, to regenerate the healthy normal tissue and avoid higher-grade side effects [23]. A further advantage of our study is the uniform fractionation scheme applied to all patients, which facilitates a reliable assessment of this fractionation.

Nair et al. [17] also compared different concepts of breast irradiation, both in the curative adjuvant setting and for palliation. In their center, irradiation with 30 Gy in five fractions was administered to the whole breast and/or nodes, once a week, followed by a 6-Gy boost to the primary breast tumor. Acceptable local control and minimum toxicity were established.

While these concepts are similar to those described in our study, the abovementioned authors did not use a simultaneous integrated boost and, consequently, used longer overall treatment times. The advantage of our radiotherapy concept is the short treatment time and the local dose increase to the primary tumor as a simultaneous integrated boost. To the best of our knowledge, this has not appeared in the literature before.

It is beneficial for elderly frail patients to complete their treatment in a short period of time. We therefore emphasize the importance of an ultra-hypofractionated approach, enabling the treatment to be completed in just one week with only five fractions. In this cohort, the overall treatment time between the first and last irradiation was 10.7 days (range 7–16 days). Many additional considerations are relevant when treating older adults in a palliative setting, such as frailty assessment and making rational treatment decisions [24].

Of all 27 patients, 9 (33%) received their first radiation treatment over 500 days after the date of initial diagnosis. These patients had already received primary anti-hormonal therapy and were irradiated with our concept in the event of local symptoms, mostly local exulceration. Of the 10 patients (37%) who were irradiated within less than 100 days of initial diagnosis, 8 had local exulceration with a high symptom burden, one was hormone receptor negative and had no options for systemic therapy due to comorbidities, and one was ineligible for systemic therapy due to general condition (ECOG 4). This explains the wide range within the patient population between initial diagnosis and first radiotherapy and shows how individually the decision is made to undergo local radiotherapy in palliative treatment regimens. The remaining patients first received systemic therapy, e.g., anti-hormonal therapy, followed by local radiation therapy in the case of symptomatic tumor burden with local symptoms.

Due to positive hormone receptors, many patients in this cohort also received systemic therapy, so the tumor responses and local control probably reflect not only the effects of local radiotherapy but also those of systemic therapies. This synergistic effect cannot be further differentiated due to the retrospective design of the study. However, since radiotherapy has been proven to be a well-tolerated temporary local treatment, it should be offered to patients as a local therapy, especially for complaints like ulceration, bleeding, and pain.

The limitations of our trial are the small number of cases and the retrospective collection of data. Furthermore, most patients had only one follow-up appointment, so late effects or re-progression were not detected in the context of our aftercare due to loss to follow-up. However, evaluating such collectives is difficult, as many patients are lost to follow-up care due to their limited general condition and the associated immobility, which makes it more difficult to assess their condition after completion of palliative radiotherapy.

## Conclusion

Treatment options for inoperable and/or frail patients with advanced breast cancer focus on symptom management in the case of pain, bleeding, or ulceration. In these cases, ultra-hypofractionated short-course irradiation is a useful option and should be offered to these patients, as it is feasible and well tolerated. It provides a high rate of local control, thus balancing risk and benefit.
